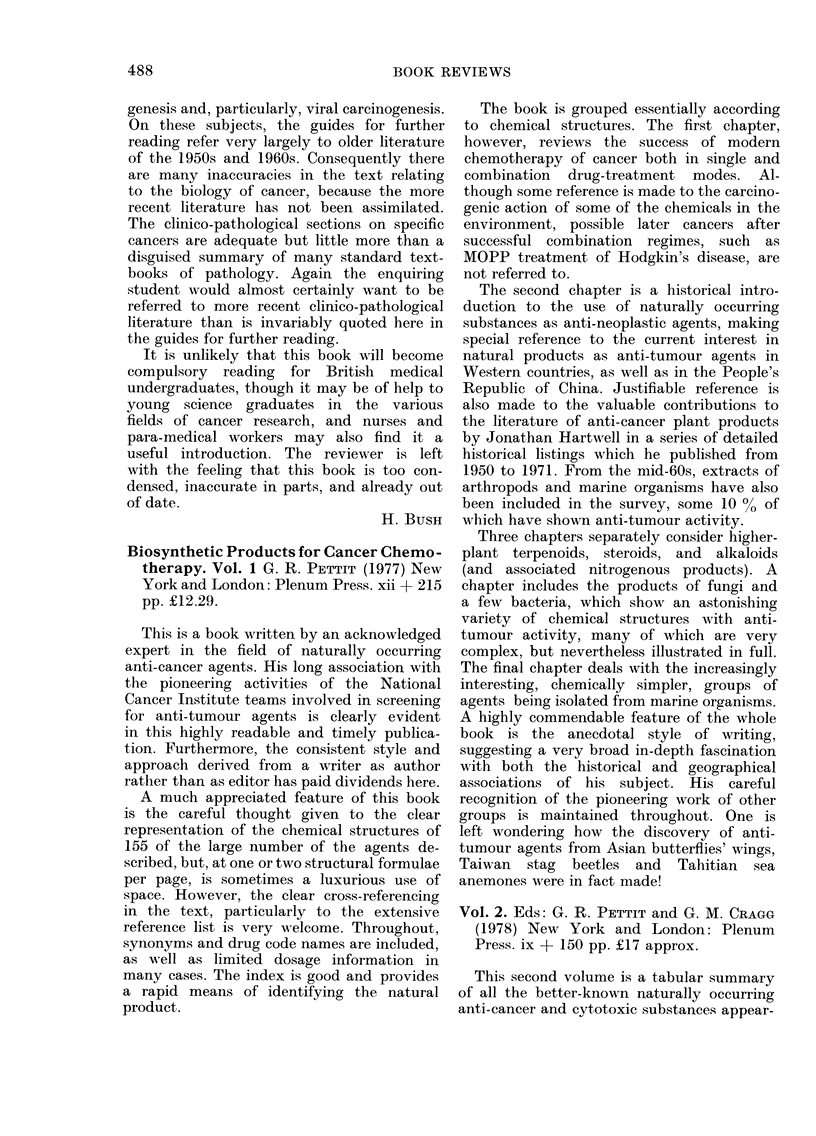# Biosynthetic Products for Cancer Chemotherapy. Vol. 1

**Published:** 1979-04

**Authors:** 


**Biosynthetic Products for Cancer Chemotherapy. Vol. 1** G. R. PETTIT (1977) New York and London: Plenum Press. xii + 215 pp. £12.29.

This is a book written by an acknowledged expert in the field of naturally occurring anti-cancer agents. His long association with the pioneering activities of the National Cancer Institute teams involved in screening for anti-tumour agents is clearly evident in this highly readable and timely publication. Furthermore, the consistent style and approach derived from a writer as author rather than as editor has paid dividends here.

A much appreciated feature of this book is the careful thought given to the clear representation of the chemical structures of 155 of the large number of the agents described, but, at one or two structural formulae per page, is sometimes a luxurious use of space. However, the clear cross-referencing in the text, particularly to the extensive reference list is very welcome. Throughout, synonyms and drug code names are included, as well as limited dosage information in many cases. The index is good and provides a rapid means of identifying the natural product.

The book is grouped essentially according to chemical structures. The first chapter, however, reviews the success of modern chemotherapy of cancer both in single and combination drug-treatment modes. Although some reference is made to the carcinogenic action of some of the chemicals in the environment, possible later cancers after successful combination regimes, such as MOPP treatment of Hodgkin's disease, are not referred to.

The second chapter is a historical introduction to the use of naturally occurring substances as anti-neoplastic agents, making special reference to the current interest in natural products as anti-tumour agents in Western countries, as well as in the People's Republic of China. Justifiable reference is also made to the valuable contributions to the literature of anti-cancer plant products by Jonathan Hartwell in a series of detailed historical listings which he published from 1950 to 1971. From the mid-60s, extracts of arthropods and marine organisms have also been included in the survey, some 10 % of which have shown anti-tumour activity.

Three chapters separately consider higher-plant terpenoids, steroids, and alkaloids (and associated nitrogenous products). A chapter includes the products of fungi and a few bacteria, which show an astonishing variety of chemical structures with anti-tumour activity, many of which are very complex, but nevertheless illustrated in full. The final chapter deals with the increasingly interesting, chemically simpler, groups of agents being isolated from marine organisms. A highly commendable feature of the whole book is the anecdotal style of writing, suggesting a very broad in-depth fascination with both the historical and geographical associations of his subject. His careful recognition of the pioneering work of other groups is maintained throughout. One is left wondering how the discovery of anti-tumour agents from Asian butterflies' wings, Taiwan stag beetles and Tahitian sea anemones were in fact made!